# Evaluation and Treatment Adherence of a Child With Atopic Dermatitis Complicated by Parental Steroid Phobia: A Case Report

**DOI:** 10.7759/cureus.81132

**Published:** 2025-03-25

**Authors:** Ryosuke Sakurai, Manabu Miyamoto, Shigemi Yoshihara

**Affiliations:** 1 Department of Pediatrics, Dokkyo Medical University, Tochigi, JPN

**Keywords:** atopic dermatitis, informed consent, janus kinase inhibitors, phobic disorders, therapeutics

## Abstract

The topical corticosteroid phobia (TOPICOP) score is a useful tool for evaluating the severity of steroid phobia. Following the development of novel topical therapies, such as delgocitinib, a Janus kinase inhibitor, the treatment options for atopic dermatitis (AD) have significantly expanded. Herein, we report the case of a 6-year-old girl with moderate AD whose treatment was complicated by her mother’s steroid phobia (TOPICOP score: 52.7). Despite detailed explanations addressing misconceptions regarding topical corticosteroids (TCS), the mother refused its use. As an alternative, we initiated treatment with delgocitinib 0.5% ointment, achieving significant clinical improvement. Within one month, the patient’s eczema area and severity index score improved from 13.0 to 7.2. We reduced the concentration of the delgocitinib ointment from 0.5% to 0.25% secondary to a decreased Eczema Area and Severity Index (EASI) score. After six months, her objective AD scores also improved. This case highlights the importance of assessing and addressing steroid phobia in parents of children with AD. Despite comprehensive education, some parents remain unwilling to use TCS, necessitating alternative treatment approaches. The use of a validated tool, such as the TOPICOP score, can help clinicians objectively assess parental concerns. In cases where TCS refusal persists, delgocitinib ointment may serve as an effective induction therapy. Given its favorable safety profile and rapid efficacy, initiating treatment with the 0.5% formulation may be a viable option for achieving early disease control in moderate to severe AD. In conclusion, evaluating parental concerns with the TOPICOP score is useful in clinical practice. When steroid phobia persists despite proper education, delgocitinib ointment offers an effective alternative, with higher concentrations potentially beneficial for more severe cases.

## Introduction

Atopic dermatitis (AD) is an inflammatory skin disease characterized by recurrent eczema and itching, affecting 15-20% of children [1,2]. The primary treatment involves the use of anti-inflammatory drugs such as topical corticosteroids (TCS). However, 66-68% of parents are resistant to topical steroid therapy, a phenomenon termed “steroid phobia” [3-5]. Steroid phobia is one of the factors triggering poor treatment adherence, resulting in poor disease control in patients with AD [6]. The topical corticosteroid phobia (TOPICOP) score can be used to evaluate the severity of steroid phobia [7]. The TOPICOP score is calculated by assigning a score of 0 to 3 to each item in the questionnaire, adding up the scores, and then dividing the total by the number of items completed [7]. The maximum score is 36, which is converted to a percentage of 0 to 100. A higher score indicates a more severe condition; however, there are no defined diagnostic criteria, so the score must be evaluated relatively. The reason why it is difficult to obtain informed consent for steroid phobia is that parents' concerns about TCS are not always unfounded [7]. Therefore, we need to have a management strategy without TCS. In recent years, novel topical drugs with new mechanisms targeting AD have become available, expanding treatment options beyond traditional TCS [1]. Delgocitinib is one of the novel inhibitors of Janus kinase (JAK), an enzyme important for the signal transduction of various cytokines [8]. JAK inhibitors interrupt the signaling pathways of cytokines such as interleukin (IL)-4, IL-13, IL-22, and IL-31 [2], which are involved in the pathogenesis of atopic dermatitis. It is believed that this inhibitory effect shows efficacy against atopic dermatitis. In clinical trials involving children, delgocitinib showed significant improvement in atopic dermatitis compared to the vehicle, and there were no safety profile issues [8]. However, delgocitinib is shown to be absorbed into the body through the skin, so it is necessary to adhere to the maximum amount (5 grams per dose) [8]. In Japan, delgocitinib ointments with formulations of 0.25% and 5% have been approved for use in children [8]. Herein, we describe the successful treatment of a case of pediatric AD using delgocitinib ointment for parental steroid phobia. Further, we discuss the importance of the evaluation of parent’s hesitation to use TCS.

## Case presentation

**Figure 1:**
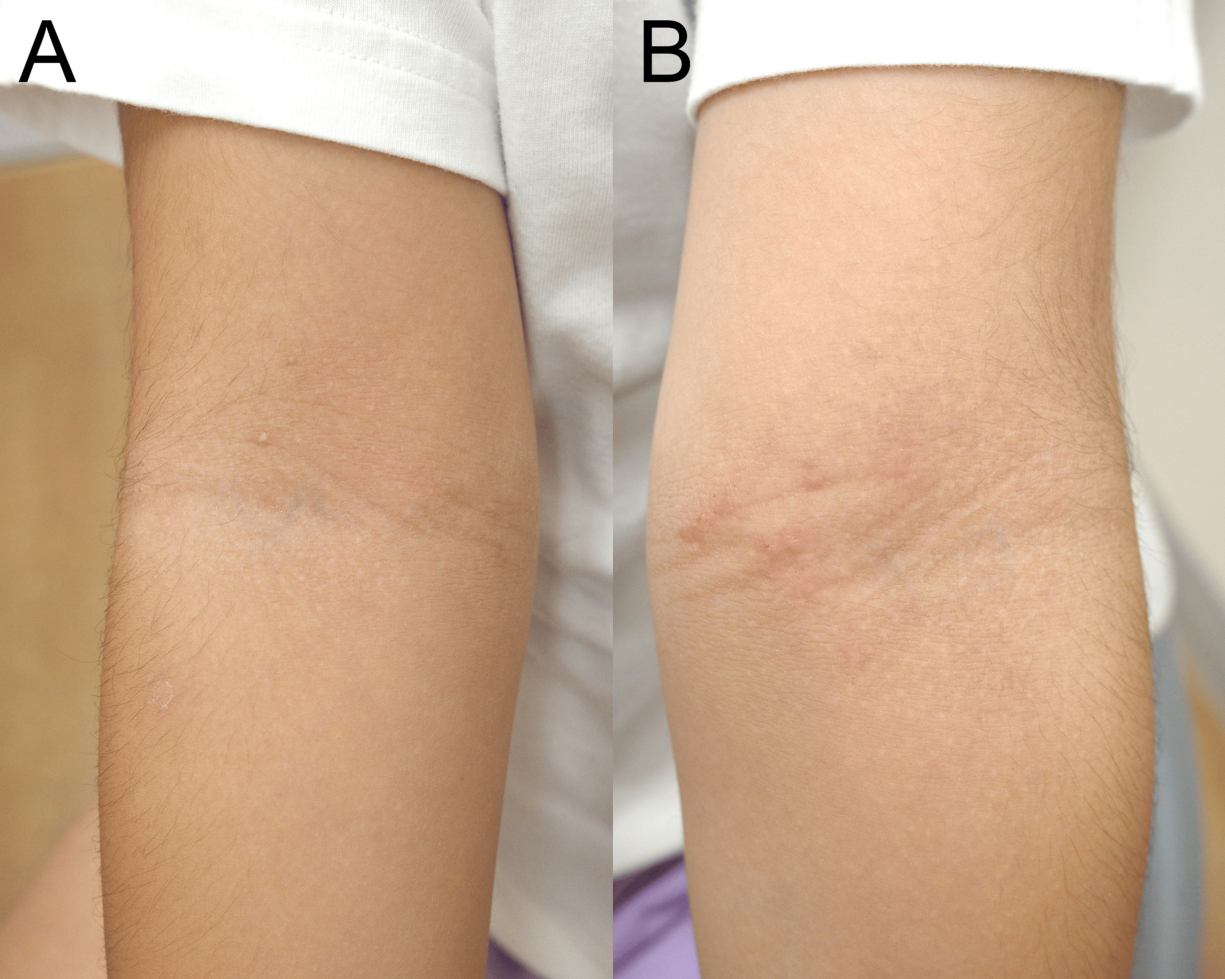
The images show the patient's right (A) and left (B) upper arms The images show the inside of the patient's right (A) and left (B) upper arm after six months of treatment with delgocitinib. There is slight erythema on the left arm. The mild residual erythema is consistent with near-complete resolution. We were not able to take a picture of the patient before treatment because we could not obtain the consent of the parents.

A six-year-old girl was referred to our hospital for treatment of AD. She had been diagnosed with AD and hen’s egg allergy at one year of age and additionally had a history of asthma, seasonal allergic rhinitis, and conjunctivitis. She had since continued treatment with a non-specialist physician, but her symptoms did not improve. She had been treated with moisturizers and antihistamines and had not used TCS even though it had been prescribed. Upon visiting our hospital, she presented with dry skin, itching, and overall skin hyperpigmentation, along with excoriations and prurigo on her lower legs. On the extremities, erythema and papulation with itching, excoriations, pigmentation, and lichenification were observed. On the face, pigmentation was observed on the eyelids. The Investigator Global Assessment for AD (vIGA-AD) scale score was 3 - moderate, and the Eczema Area and Severity Index (EASI) score was 13.0. The blood test showed that eosinophils were elevated to 880/µL, and the total IgE level was elevated to 4590 IU/mL. We recommended the use of a steroid ointment; however, her mother declined, citing previous side effects, including depigmentation, from steroid treatment for her own AD. The mother’s TOPICOP score was 52.7 (Table 1). Based on her TOPICOP score, we hypothesized that the mother's concerns regarding TCS use predominantly stemmed from anxieties about TCS. Therefore, we explained that the appropriate use of TCS would minimize side effects and maximize efficacy. Moreover, the side effects she experienced were likely a misunderstanding; however, we could not persuade the mother to agree to TCS use. Therefore, we explained general information about delgocitinib ointment, including the side effects, to the patient's mother. In other words, the amount of drug used per treatment should be limited to 5 grams, and it should be applied to the lesions twice a day. Delgocitinib ointment 5% is approved for use in children, however, if the symptoms improve, the ointment should be changed to 0.25%. If there are any symptoms of folliculitis, local skin infection, acne, or contact dermatitis, which may be side effects, the use of the ointment should be discontinued. After receiving our explanation, the mother agreed to use delgocitinib. We, therefore, prescribed a moisturizer and delgocitinib 0.5% ointment twice a day for the patient. After one month, although the prurigo on the patient’s lower limbs persisted, her EASI score improved to 7.2. She did not develop any side effects such as infections or acne. We reduced the concentration of the delgocitinib ointment from 0.5% to 0.25% secondary to the decreased EASI score. Both the patient and her mother were optimistic about the continued use of delgocitinib ointment and successfully continued treatment. We assessed the patient's condition every one to two months and checked the use of the ointment at each assessment. Subsequent skin improvement was observed, and after six months, the patient’s vIGA-AD and EASI scores were 1 and 1.3, respectively (Figures 1A, 1B). After we confirmed that her skin condition had improved, we scheduled an oral food challenge test with a hen’s egg.

**Table 1:** TOPICOP score of our patient's mother The table shows the answers of our patient's mother related to the TOPICOP score. This score is calculated by assigning a score of 0 to 3 to each item in the questionnaire, adding up the scores, and then dividing the total by the number of items completed. The maximum score is 36, which is converted to a percentage of 0 to 100. The mother’s TOPICOP score was 52.7. Higher scores correlate with greater severity of steroid phobia. TCS: topical corticosteroids; TOPICOP: topical corticosteroid phobia

	Item	The mother's response
1	TCS pass into the bloodstream	Not really agree
2	TCS can lead to infections	Not really agree
3	TCS make you fat	Not really agree
4	TCS damage your skin	Totally agree
5	TCS will affect my future health	Almost agree
6	TCS can lead to asthma	Not really agree
7	I don’t know of any side effects but I’m still afraid of TCS	Almost agree
8	I’m afraid of applying too much cream	Often
9	I’m afraid of putting cream on certain zones like eyelids, where the skin is thinner	Often
10	I wait as long as I can before treating myself	Sometimes
11	I stop the treatment as soon as I can	Sometimes
12	I need reassurance about TCS	Often

## Discussion

In our case, we were unable to change the patient's mother's attitude to TCS, despite evaluating the mother’s fear of TCS using the TPICOP score and providing full explanations of the effectiveness and possible adverse effects of TCS. It has been pointed out that is important to spend time assessing parents' anxieties and worries about TCS to eliminate parents' fears of TCS [4]. Furthermore, providing sufficient education for the patient and the parents can improve feelings of aversion to TCS and reduce the patient’s disease burden [9]. It may further be useful to use a self-administered questionnaire, such as the TOPICOP score, to evaluate the parents’ feelings of aversion to TCS. We evaluated the TOPICOP score when we felt that the parents had concerns about TCS; however, it may be necessary to evaluate it each time TCS is prescribed ideally. In one prior cross-sectional study of AD patient’s parents in Japan, the average TOPICOP score was 40.3, with higher scores observed among parents of patients with moderate or severe AD [10]. A similar survey conducted internationally identified factors contributing to steroid phobia, including prior negative experiences with TCS and the patient’s female gender. Our patient exhibited some of these factors, and her mother's TOPICOP score was higher than the Japanese average. This case suggests that if a patient's parent has a high TOPICOP score and refuses treatment despite our thorough explanation, Janus kinase inhibitor ointment may be considered as an induction therapy.

In Japan, delgocitinib ointments in formulations of 0.25% and 5% are approved for use in children, with the 0.25% formulation generally recommended as the first-line treatment based on clinical trial data [11]. However, if symptoms are severe and the 0.25% formulation proves insufficient, the 0.5% formulation can be used [11]. In clinical trials, delgocitinib ointment achieved improvement in AD children after four weeks of treatment [12]. We initially used the 5% formulation based on the patient’s moderate disease severity. In the trials, there was little difference in improvement between the 0.25% and 0.5% formulations in children with moderate to severe AD (IGA 3-4) [12]; thus, it may have been acceptable to use the 0.25% formulation for the initial treatment of this patient. However, because our patient had not received anti-inflammatory treatment before treatment, we used the more effective 0.5% formulation to induce early remission, and it was effective within four weeks. The patient's parents appreciated seeing positive results within one month. Previous reports have shown no significant differences in adverse reactions between the 0.25% and 5% formulations in pediatric patients [12], suggesting that starting treatment with the 5% formulation is a viable option.

The limitations of this report are as follows. First, because the patient's course was short, at only six months, long-term follow-up data are absent. Second, because JAK inhibitors are a new mechanism of the drug, data on the effects of systemic absorption and long-term treatment risks in children are missing. Third, to determine whether the results obtained for our patient can be used regardless of race or severity of steroid phobia, further data are needed.

## Conclusions

Based on this case, we believe that using an objective scoring system, such as the TOPICOP score, to assess the concerns of parents of children with atopic dermatitis is useful in evaluating the attitude to TCS. However, if parents are still hesitant to use TCS even after being provided sufficient information regarding TCS, we would consider it acceptable to start treatment with a new anti-inflammatory drug containing delgocitinib.

## References

[1] Langan SM, Irvine AD, Weidinger S (2020). Atopic dermatitis. Lancet.

[2] Schuler CF 4th, Billi AC, Maverakis E, Tsoi LC, Gudjonsson JE (2023). Novel insights into atopic dermatitis. J Allergy Clin Immunol.

[3] Lee JY, Her Y, Kim CW, Kim SS (2015). Topical corticosteroid phobia among parents of children with atopic eczema in Korea. Ann Dermatol.

[4] Albogami M, AlJomaie M, Almarri S (2023). Topical corticosteroid phobia among parents of children with atopic dermatitis (eczema) - a cross-sectional study. Patient Prefer Adherence.

[5] Salas-Walinsundin WM, Wong V, Chong JH, Koh MJ (2020). Steroid phobia in children with atopic dermatitis and their caregivers in Singapore. Dermatol Ther.

[6] Sokolova A, Smith SD (2015). Factors contributing to poor treatment outcomes in childhood atopic dermatitis. Australas J Dermatol.

[7] Moret L, Anthoine E, Aubert-Wastiaux H (2013). TOPICOP©: a new scale evaluating topical corticosteroid phobia among atopic dermatitis outpatients and their parents. PLoS One.

[8] Saeki H, Ohya Y, Arakawa H (2025). English version of clinical practice guidelines for the management of atopic dermatitis 2024. J Dermatol.

[9] Wilken B, Zaman M, Asai Y (2023). Patient education in atopic dermatitis: a scoping review. Allergy Asthma Clin Immunol.

[10] Saito-Abe M, Futamura M, Yamamoto-Hanada K, Yang L, Suzuki K, Ohya Y (2019). Topical corticosteroid phobia among caretakers of children with atopic dermatitis: a cross-sectional study using TOPICOP in Japan. Pediatr Dermatol.

[11] Fukuie T, Toyama H, Tanaka M, Ohashi-Doi K, Kabashima K (2024). Post-hoc safety/efficacy analyses from pediatric delgocitinib atopic dermatitis trials. Pediatr Int.

[12] Nakagawa H, Nemoto O, Igarashi A, Saeki H, Oda M, Kabashima K, Nagata T (2019). Phase 2 clinical study of delgocitinib ointment in pediatric patients with atopic dermatitis. J Allergy Clin Immunol.

